# Editorial: Modern Management and Monitoring of Childhood Diabetes

**DOI:** 10.3389/fendo.2022.900278

**Published:** 2022-04-25

**Authors:** Valentino Cherubini, Valentina Tiberi, Francesco Chiarelli

**Affiliations:** ^1^Department of Women’s and Children’s Health, “G. Salesi” Hospital, Ancona, Italy; ^2^Department of Pediatrics, University of Chieti, Chieti, Italy

**Keywords:** childhood diabetes, management – healthcare, epidemiology of diabetes, diabetes retinopathy, prediction of diabetes, DKA (diabetic ketoacidosis)

Incidence and prevalence of type 1 diabetes in youth are increasing worldwide, while the disease prevention progress slower than hoped. It is estimated that the incidence of type 1 diabetes among children and adolescents under the age of 15 years increase around 3% with strong geographic differences. Therefore, the management and monitoring of childhood diabetes play a central role in clinical practice, which cannot simply derive from the knowledge gained from the management of adults with diabetes. Epidemiology, diagnosis and therapy, as well as implications on growth and impact on the family, require specific pediatric management.

The purpose of this Research Topic is to provide an update on modern diabetes management and monitoring in young people, focusing on prediction, epidemiology, treatment, technology and complications.

The increase in the frequency of diabetic ketoacidosis (DKA) in newly diagnosed children with type 1 diabetes does not appear to stop in most countries, including those with a free healthcare system. Only in Italy was a slight reduction in DKA at the onset of diabetes in the 2014-2016 period observed (Gesuita et al.). This study also found that socioeconomic inequalities, measured as low levels of education and employment, were associated with a higher likelihood of DKA at the onset of type 1 diabetes. Continuous monitoring of the frequency of DKA is of utmost importance for the prevention of this fearful complication in children. On the other hand, the prevention of this terrible complication is possible through targeted information campaigns and through population screening for type 1 diabetes. Currently available genetic, immunological and metabolic biomarkers allow to characterize children at high risk of progression to type 1 diabetes, which has stimulated numerous studies on secondary and tertiary prevention. The challenge, still open, is to preserve the residual destruction of beta cells and/or prolong the phase of remission after the onset of type 1 diabetes. In most cases, type 1 diabetes is diagnosed in children due to the presence of characteristic symptoms of hyperglycemia such as intense thirst and increased urine output. Identifying patients before the onset of disease symptoms is an opportunity to prevent DKA, which is associated with permanent neurological damage, high biological and social costs, and poor long-term metabolic control. In this Research Topic is included a review focusing on the main current knowledge on the prediction and prevention of type 1 diabetes in children (Primavera et al.).

Once diagnosed with type 1 diabetes, regular self-monitoring of blood glucose and ketone levels, when indicated, is an essential component of managing type 1 diabetes. The use of fingerstick glucose monitoring has been considered the standard of cure for decades, now giving way to continuous glucose monitoring (CGM) with the glucose sensor. Marks and Wolfsdorf thoroughly review recommendations for self-monitoring of glucose and ketones in pediatric T1D with particular emphasis on CGM and the factors affecting the accuracy and real-world use of this technology.

Although there has been a general improvement in metabolic control in children around the world in recent years, in some countries the HbA1c values ​​in young people with type 1 diabetes are even higher than in others. The average HbA1c of young people in the United States is higher than much of the developed world. A team from Stanford University, California, USA (Prahalad et al.), including physicians, nurses, certified diabetes educators, dieticians, social workers, psychologists and exercise physiologists, proposed a diabetes education program structured by the diagnosis, using a methodology called the 4T approach: teamwork, goals, technology and tight control. Part of the educational curriculum involves early integration of technology, particularly continuous glucose monitoring (CGM) and the development of a curriculum on using CGM to maintain tight control and optimize quality of life.

Severe hypoglycemia (SH) in pediatric patients with type 1 diabetes is decreased during last decades but still remains a common problem and is the main barrier to achieving good metabolic control (Urakami). Recent data showed a trend towards a marked reduction in the incidence of SH in the post-DCCT setting (from 62.0 per 100 patient-years to 1.21-30 per 100 patient-years). Advanced technologies, such as Continuous Glucose Monitoring (CGM), Sensor-Advanced Pump Therapy (SAP), Predictive Low Glucose Management (PLGM), Hybrid Closed Loop (HCL) and Advanced Hybrid Closed Loop (AHCL) systems reduce potentially minimizing the onset of severe hypoglycemia without worsening overall glycemic control. The use of advanced technologies has been shown to be able to maintain glucose in normal ranges in children during sleep, physical activity and special conditions such as surgery (Dominguez-Riscart et al.).

Although the new advances have improved glycemic control and reduced hypoglycemia, long-term complications are still an ongoing burden for patients with diabetes, and 1 in 3 young people with type 1 diabetes have at least one diabetes complication. diabetes (Graves and Donaghue). Risk factors for diabetes complications begin to manifest in childhood and adolescence, and in some young people, chronic complications may be diagnosed before moving on to adult care. This Research Topic discusses the prevalence, risk factors, screening, and treatment recommendations for vascular complications in children and adolescents with diabetes.

A recently proposed biological marker for chronic complications of diabetes is the Klotho protein. It is a transmembrane protein also present in a soluble form in the blood, mainly present in human brain, kidney, vascular tissue and other endocrine organs. The KLOTHO gene was identified as an ageing suppressor gene that extends life span when overexpressed and accelerates ageing when disrupted. Zubkiewicz-Kucharska et al. studied the serum concentration of Klotho in children with diabetes, reporting its possible involvement in the development of chronic complications.

## Author Contributions

VC, VT, and FC conceived the study, VC drafted the manuscript. All authors revised and approved the final version of this report.

## Conflict of Interest

The authors declare that the research was conducted in the absence of any commercial or financial relationships that could be construed as a potential conflict of interest.

## Publisher’s Note

All claims expressed in this article are solely those of the authors and do not necessarily represent those of their affiliated organizations, or those of the publisher, the editors and the reviewers. Any product that may be evaluated in this article, or claim that may be made by its manufacturer, is not guaranteed or endorsed by the publisher.

